# Supplementary material for: Pan-tissue mitochondrial phenotyping reveals lower OXPHOS expression and function across cancer types
Source: Sci Rep. 2023 Oct 5;13:16742. doi: 10.1038/s41598-023-43963-5 (PMC10556099; doi:10.1038/s41598-023-43963-5)
Supplement: Supplementary file 1 — Supplementary Legends. [file 41598_2023_43963_MOESM1_ESM.docx]

**Pan-tissue mitochondrial phenotyping reveals lower OXPHOS expression and function across cancer types**

Ilya N Boykov^1,2,#^, McLane M Montgomery^1,2,#^, James T Hagen^1,2^, Raphael T Aruleba^1,2^, Kelsey L McLaughlin^1,2^, Hannah S Coalson^1,2^, Margaret A Nelson^1,2^, Andrea S. Pereyra^1,2^, Jessica M. Ellis^1,2^, Tonya N Zeczycki^3^, Nasreen A Vohra^4^, Su-Fern Tan^5^, Myles C. Cabot^2,3^, Kelsey H. Fisher-Wellman^1,2,6,^*

^1^Department of Physiology, Brody School of Medicine, East Carolina University, Greenville, NC

^2^East Carolina Diabetes and Obesity Institute, East Carolina University, Greenville, NC

^3^Department of Biochemistry and Molecular Biology, Brody School of Medicine, East Carolina University, Greenville, NC

^4^Department of Surgery, Brody School of Medicine, East Carolina University, Greenville, NC

^5^Department of Medicine, Division of Hematology/Oncology, University of Virginia School of Medicine, Charlottesville, VA

^6^UNC Lineberger Comprehensive Cancer Center, University of North Carolina at Chapel Hill School of Medicine, Chapel Hill, NC, US

^#^Authors contributed equally

* To whom correspondence should be addressed:

Kelsey H. Fisher-Wellman [fisherwellmank17@ecu.edu](mailto:fisherwellmank17@ecu.edu)

East Carolina Diabetes and Obesity Institute, 115 Heart Drive, Greenville, NC 27834 USA

Telephone: 252-744-2585

**Supplementary Material:**

**Supplemental Table 1. Proteomics Data**. (**A**) OXPHOS complex and dehydrogenase database. (**B**) Analyzed results for Figure 1 – BAT, HRT, KID. (**C**) Analyzed results for Figure 1 – LIV (Tumor) and LIV (Normal). (**D**) Analyzed results for Figure 1 – COL (Normal), includes data from normal colon tissue, as well as isolated mitochondria from normal colon. (**E**) Analyzed results for Figure 1 – COL (Tumor). (**F**) Analyzed results for Figure 2 – Permeabilized tissues (Colon, CT26.WT, Heart). (**G**) Analyzed results for Figure 4 – AML (C1498), BMMC.

**Supplemental Table 2. Source Data**. Individual data points for all figures.
